# PD18 Challenges In Evaluating An Artificial Intelligence-Based Healthcare Application For Enhancing Breast Cancer Locoregional Treatment Decisions: The CINDERELLA Project

**DOI:** 10.1017/S0266462325101530

**Published:** 2025-12-29

**Authors:** Ludovica Borsoi, Oriana Ciani, Elisabetta Listorti, Natalia Oprea

## Abstract

**Introduction:**

The ongoing CINDERELLA trial evaluates an innovative artificial intelligence (AI)-based tool designed to enhance the shared decision-making process in breast cancer treatment. The trial aimed to assess the effectiveness of the CINDERELLA app in improving patient satisfaction with locoregional treatment aesthetic outcomes and evaluate its influence on overall quality of life and psychological well-being, as well as its economic, organizational, and environmental impacts.

**Methods:**

An international, multicenter randomized controlled, open-label trial is currently recruiting patients with primary early stage breast cancer to follow either a comprehensive AI-powered approach with photographs of aesthetic outcomes of various surgical techniques or standard of care. A trial-based economic evaluation from a societal perspective is planned together with a budget impact analysis. The implementability of the CINDERELLA approach was assessed with respect to its usability, acceptability, organizational impact, and overall feasibility. The environmental impact was quantitatively assessed across several dimensions, such as quantity, appropriateness, and emissions, supplemented by qualitative insights. Overall, data were gathered from questionnaires, interviews, focus groups, and app data.

**Results:**

The clinical trial has recruited 1,000 patients across seven study sites in five countries. So far, the average duration of app use was 19.25 minutes (median four logins/user). There were no statistically significant differences in median app usage time based on age group (p=0.136) or estimated type of surgery (p=0.721). The focus groups with healthcare professionals and managers across different sites highlighted comparative similarities and differences across organizational models that will aid interpretation of trial results and adaptation of the intervention to different settings.

**Conclusions:**

Initial data indicated good engagement with the CINDERELLA app. The presence of only minor differences in usage patterns across countries, age groups, and treatment types suggested it can fill information gaps for patients with breast cancer. A multidimensional evaluation of the CINDERELLA app can foster its successful translation into real-world settings, hopefully benefiting patients and clinical practice.

